# Incidence and prevalence of clinically detected smoldering multiple myeloma within the general population: a retrospective observational cohort study

**DOI:** 10.1038/s41408-025-01352-3

**Published:** 2025-08-29

**Authors:** Adrian Wong, Victor H. Jimenez-Zepeda, Kathryn Rankin, Irwin Sandhu, Michael Chu, Aurélien Delluc, Hira Mian, Julie Stakiw, Christopher Cipkar, Arleigh McCurdy, Benjamin Patrick, Sarah Albert, Meriem Henia, Edward Koo, Hyra Sapru, Christopher McCudden, Alissa Visram

**Affiliations:** 1 University of Ottawa, Ottawa, ON Canada; 2 Arnie Charbonneau Cancer Institute, University of Calgary, Calgary, AB Canada; 3 Department of Oncology, Cross Cancer Institute, University of Alberta Edmonton, Ottawa, AB Canada; 4 Division of Hematology, The Ottawa Hospital, Ottawa, ON Canada; 5 Department of Oncology, McMaster University, Hamilton, ON Canada; 6 Saskatoon Cancer Clinic, Saskatoon, SK Canada; 7 Department of Pathology and Laboratory Medicine, The Ottawa Hospital, Ottawa, ON Canada

**Keywords:** Myeloma, Cancer epidemiology

Smoldering multiple myeloma (SMM) is a precursor plasma cell disorder characterized by clonal proliferation without end-organ damage. Although asymptomatic, SMM remains clinically relevant due to its potential progression to multiple myeloma (MM) or AL amyloidosis. Recent studies suggest that early therapeutic intervention may delay progression in high-risk cases [1–4]. However, the real-world epidemiology of SMM—particularly cases diagnosed through routine clinical evaluation rather than screening—remains poorly characterized. Population-based screening, such as the iStopMM study, identified a 0.53% prevalence of SMM in individuals aged ≥40 years [5]. But these estimates may differ in clinical practice, where SMM is typically diagnosed incidentally during evaluation for other conditions. Prior studies using administrative databases have been limited by the absence of an ICD code to differentiate SMM from untreated or “SLiM” MM [6–8], making it difficult to assess true population-level trends. We aimed to address this gap by describing the incidence and prevalence of clinically detected SMM between 2010 and 2022 using real-world data from a defined Canadian health region. We conducted a retrospective cohort study using laboratory and clinical data from The Ottawa Hospital, the sole tertiary hematology center for Ontario’s Champlain Local Health Integration Network (LHIN). Due to the regionally centralized healthcare delivery and universal healthcare model in Ontario, all patients within the Champlain LHIN suspected to have a malignant hematologic disorder requiring a bone marrow biopsy—such as SMM—must be referred to our institution. In contrast, MGUS may be diagnosed and monitored by community hematologists or internists and would not necessarily be captured in our dataset. We identified all adults tested for monoclonal proteins (serum protein electrophoresis [SPEP], urine protein electrophoresis [UPEP], serum free light chains [FLC], and immunofixation) between January 1, 2010, and December 31, 2022. We flagged patients with detectable monoclonal proteins (MCP) or abnormal FLC ratios and cross-referenced pathology records for bone marrow biopsies. We also retrieved treatment data from the Ontario Cancer Registry to identify patients who received therapies indicative of plasma cell or lymphoproliferative disorders (see supplementary data for further details). Electronic medical records were reviewed in detail to discern the workup and diagnosis of patients. We defined SMM as either: (i) ≥10% bone marrow plasma cells (BMPCs) without CRAB or SLiM criteria, or (ii) MCP ≥ 30 g/L without MM-defining events [9]. We applied the Mayo 20/20/20 risk model to classify patients as high-risk if they had ≥2 of the following: BMPCs >20%, MCP > 20 g/L, or FLC ratio >20 [10]. As SMM is asymptomatic the true incidence of SMM cannot be determined, as this would require screening for MGUS and monitoring for progression to SMM. Therefore, incident SMM was defined as the date that a patient was first diagnosed with SMM during clinical evaluation. We defined incidence as the number of new SMM diagnoses per year and prevalence as the number of alive, non-progressed, and actively followed SMM patients in a given year (even if diagnosed previously). Publicly reported Champlain LHIN census data from 2011, 2016, and 2021 were used to calculate incidence and prevalence rates of SMM within the general population [11–13]. We evaluated 51,798 patients for monoclonal gammopathies over the study period, of whom 7,431 (14.3%) had a detectable MCP. Among these, 344 patients had confirmed SMM. Figure S1 outlines the full cohort selection. Of the 344 patients, 260 were diagnosed during the study period (incident cases), while the rest were diagnosed before 2010 but remained under follow-up. The median age at diagnosis was 70.9 years (IQR 61.8–79.4), and 53% were male. Median MCP was 12.4 g/L (IQR 6.4–22.2), and the median FLC ratio was 9.5 (IQR 3.1–28.1). Only 3 patients were diagnosed before age 40. Table 1 summarizes baseline characteristics stratified by diagnostic period. We observed improved diagnostic completeness over time: by 2020–2022, 99% of patients had both a bone marrow biopsy and FLC evaluation, compared to 0% having a FLC and only 77% undergoing a baseline bone marrow biopsy in 2010–2014. The number of new SMM diagnoses in patients above 40 years rose from 14 in 2011 to 28 in 2022. Incidence rates per 100,000 Champlain LHIN residents increased from 0.7 in 2011 (0.0007% of the population) to 1.9 (0.0019% of the population) in 2021. Among individuals aged ≥40, incidence increased from 1.5 to 3.6 per 100,000 people over the same period. We performed age-specific standardization using the 2016 population as reference; the standardized incidence ratio (SIR) in 2021 was 1.9 (95% CI 1.2–2.6), indicating that observed cases were nearly double the expected (compared to the 2016 incidence rates). Tables S1, S2 summarizes these age-specific SIRs and incidence stratified by age and sex over time, respectively. Importantly, the increase in SMM incidence appeared driven by low- and intermediate-risk patients. Figure 1B shows that while overall incidence increased, the proportion of high-risk SMM remained stable. Table 1 supports this trend, showing a significant decline in median MCP over time (2010–2014: 15.0 g/L vs. 2020–2022: 9.2 g/L, *p* < 0.001).

**Table 1:** Baseline characteristics of clinically detected smoldering multiple myeloma patients diagnosed between 2010 and 2022.

	Date of SMM diagnosis			Total SMM cohort (n = 260)
	2010-2014 (n = 69)	2015-2019 (n = 110)	2020-2022 (n = 81)	
Median age at SMM diagnosis—n (IQR)	71.6 (62.5–80.7)	70.6 (61.2–79.7)	71.3 (60.9–78.7)	70.9 (61.8–79.4)
Male sex—n (%)	34 (49)	61 (55)	44 (54)	139 (53)
Median MCP (g/L)—n (IQR)	15 (9.2–27.8)	14.4 (7.1–23.1)	9.2 (3.6–17.1)	12.4 (6.4–22.2)
Median BM PC percentage—n (IQR)	15 (10–30)	15 (10–30)	20 (12–25)	15 (11–30)
Median FLC ratio^a^—n (IQR)	-	12.1 (3.9–32.0)	7.6 (2.7–21.7)	9.5 (3.1–28.1)
Heavy Chain isotype—n (%)				
IgG	53 (77)	79 (72)	51 (63)	183 (76)
IgA	11 (16)	17 (15)	20 (25)	48 (20)
IgM	1 (1)	1 (1)	2 (2)	2 (1)
None or missing	3 (4)	13 (12)	8 (10)	7 (3)
Light chain isotype—n (%)				
Kappa	44 (64)	65 (59)	56 (69)	165 (63)
Lambda	22 (32)	42 (38)	22 (27)	86 (33)
Missing	3 (4)	3 (3)	3 (4)	9 (3)
Diagnostic workup—n (%)				
FLC testing	0	90 (82)	80 (99)	170 (65)
BM biopsy	53 (77)	106 (96)	80 (99)	239 (92)
Cross-sectional imaging^b^	24 (35)	83 (75)	65 (80)	172 (66)

*SMM* smoldering multiple myeloma, *MCP* monoclonal protein, *IQR* interquartile range, *BM PC* bone marrow plasma cell, *FLC* free light chain ratio.

^a^FLC of involved to uninvolved free light chain.

^b^Cross-sectional imaging was defined as MRI spine whole spine with spine and pelvis, or MRI axial skeleton, or CT chest and abdomen and pelvis, or PET/CT scan.

**Fig. 1:**
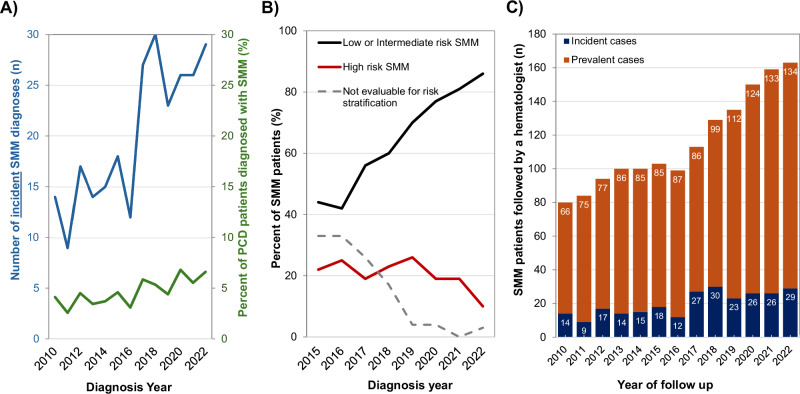
Incidence and prevalence of SMM over time. **A** Incidence of annual SMM cases in general over time, as well as among patients evaluated for a plasma cell disorder (PCD). **B** SMM incidence stratified by the baseline Mayo 20/20/20 risk score *(patients with missing BM biopsy, FLC ratio, or serum MCP data were deemed non-evaluable for risk stratification)*. **C** Prevalence of SMM over time. Total prevalent SMM is presented, stratified by annual incident and prevalent cases.

SMM prevalence also increased over time, rising from 6.8 per 100,000 residents in 2011 to 11.4 in 2021. Among adults ≥40 years, prevalence rose from 13.4 to 21.8 per 100,000. We observed increasing prevalence with age: in 2021, prevalence was 3.4 per 100,000 among patients aged 40–49, compared to 71.8 per 100,000 among those ≥80. Table S3 details these trends by age and sex. The prevalence of SMM was consistently higher among older patients; in 2021, among residents aged 40–49 versus ≥80 years, the SMM prevalence was 3.5 versus 71.8 cases per 100,000 residents (SMM prevalence of 0.003% versus 0.07%, respectively). Again, we emphasize that prevalence reflects a cumulative count of living, actively followed, and non-progressed patients, not just those newly diagnosed in a given year.

The increased incidence of SMM diagnoses over time is most likely due to earlier detection of lower-risk disease. This is supported by laboratory testing patterns, whereby Fig. S2 shows that the number of SPEP and UPEP tests remained relatively stable over time, while FLC testing increased steadily. This likely reflects expanded test availability in recent years. Increased bone marrow biopsy use may also have been driven by the 2014 IMWG revision of MM criteria, which reclassified ultra high-risk SMM (“SLiM”) patients as having MM [9], and by growing evidence supporting risk stratification of SMM to guide monitoring or early therapeutic intervention strategies.

Despite these increasing incidence of clinically-detected SMM, the prevalence of clinically detected SMM remains lower than expected. For instance, in the iStopMM study, 0.53% of screened adults ≥40 years had SMM [5], whereas we observed only 0.02% among the same age group. This discrepancy likely reflects underdiagnosis of SMM in the real-world setting. In real-world practice, clinicians may avoid invasive testing for patients with low-risk MGUS, especially when therapeutic implications are limited. Furthermore, in Ontario, treatment for high-risk SMM is not routinely reimbursed, possibly reducing motivation to pursue definitive diagnostic workups. The underdiagnosis of low-risk SMM is likely reflected in other clinical cohorts, where the median MCP at diagnosis typically ranges from 18–20 g/L [10, 14]—notably higher than the 6.2 g/L median observed in the iSTOP-MM cohort [5], which rigorously followed IMWG guidelines for performing bone marrow biopsies.

Our study has several strengths. The Ottawa Hospital serves as the sole malignant hematology referral center for the Champlain LHIN, ensuring that all patients suspected to have SMM who require diagnostic bone marrow biopsy are referred. This makes our dataset uniquely suited for population-based estimates. We performed manual chart review to distinguish SMM from MGUS and MM, overcoming the limitations of prior administrative database studies which relied on algorithms combining ICD disease classification codes and treatment data to infer whether patients had SMM [6–8]. We also included all patients evaluated for MCPs, minimizing selection bias. Nonetheless, we acknowledge certain limitations. Community-diagnosed high-risk MGUS patients not referred for further evaluation, or MGUS patients with evolving MCPs that did not undergo repeat bone marrow biopsy would not have been re-classified as SMM, which likely underestimates true SMM prevalence. Given the variability in access to investigations and the change in definition of MM over time, it is possible that some SMM patients early in the study follow-up may have actually had SLiM MM and were misclassified as SMM. Lastly, our findings may not generalize to jurisdictions with broader access to testing or where SMM treatment is reimbursed. In conclusion, we demonstrate that the incidence and prevalence of clinically detected SMM have increased over the past decade in Ontario. However, this increase is largely driven by improved diagnostic evaluation and earlier detection of lower-risk cases, rather than an increase in high-risk disease. The prevalence of SMM remains far below that observed in screening studies, underscoring the likelihood of missed or undiagnosed cases in routine practice. These findings highlight the need to align diagnostic practices with evolving criteria and to explore the implications of earlier detection for patient outcomes and health system planning.

## Supplementary information

Supplementary Material (Clinically detected SMM incidence and prevalence over time)
