# Supplementary material for: Incidence and prevalence of clinically detected smoldering multiple myeloma within the general population: a retrospective observational cohort study
Source: Blood Cancer J. 2025 Aug 29;15(1):149. doi: 10.1038/s41408-025-01352-3 (PMC12397400; doi:10.1038/s41408-025-01352-3)
Supplement: Supplementary file 1 — Supplementary Material (Clinically detected SMM incidence and prevalence over time) [file 41408_2025_1352_MOESM1_ESM.docx]

**Supplementary Appendix**

**Detailed study methodology**

**Population-level data**Prior to 2023, the delivery of publicly funded healthcare services in Ontario, Canada were divided between 14 geographically distinct local health integration networks (LHIN) (10). The Ottawa Hospital is the only tertiary care hospital within the Champlain LHIN, and therefore all patients within the Champlain LHIN requiring assessment or treatment by a malignant hematologist are evaluated at our center. Therefore, the cases of SMM evaluated at the Ottawa Hospital can be assumed to reflect the SMM cases within the Champlain LHIN region. As such, to determine the incidence and prevalence rates of SMM within the general population, the population of the Champlain LHIN was used as the reference. Demographics and population size of the Champlain LHIN were obtained from publicly available Census data from 2011, 2016 and 2021 (11-13).

**Clinical Population**

Electronic medical records were used to identify patients that had a serum protein electrophoresis (SPEP), urine protein electrophoresis (UPEP), urine immunofixation (uIFE), serum immunofixation (sIFE), or serum free light chain (FLC) assay ordered between January 1, 2010 and December 31, 2022 as part of routine clinical care at the Ottawa Hospital. Patients with a detectable serum or urine monoclonal protein (MCP) or an abnormal FLC ratio (defined as <0.26 or >1.65) (14) were identified. All available serial SPEP, UPEP, and SFLC laboratory results were retrieved between the first date of a documented MCP, even if prior to January 1, 2010, and the date of last study follow-up, which was December 1, 2023 to ensure at least 1 year of follow up. Electronic medical records were then used to assess whether patients with an identified MCP had a bone marrow biopsy pathology report. Similarly, the Ontario Cancer Registry was used to identify whether patients received at least 1 dose of a regimen containing daratumumab, lenalidomide, bortezomib, melphalan, cyclophosphamide, bendamustine, rituximab, vincristine, fludarabine, chlorambucil, ibrutinib, daunorubicin, or doxorubicin. Patients receiving treatment regimens containing these medications were considered to have been possibly treated for a lymphoproliferative or plasma cell disorder. Given that Canada has a publicly funded universal healthcare system which provides access to healthcare expenditures including chemotherapy agents, all patients treated with publicly reimbursed regimens for approved indications would be included in this list. Similarly, as the Ottawa Hospital is the only malignant hematology tertiary care center within the Champlain LHIN, all patients treated for a lymphoproliferative or plasma cell disorder in the region would have been treated at this center and would have been identified in this data pull.

Using the serial laboratory, pathology, and treatment data, we assumed that patients with a maximum MCP during follow up of <10 g/L, and a maximum serum FLC ratio <10, and no bone marrow biopsy report, and no recorded exposure to plasma cell or B-cell directed therapy were most likely patients with MGUS that did not progress to a lymphoproliferative or plasma cell disorder. For all patients that did not meet these criteria, medical charts were manually reviewed to identify the date and type of first lymphoproliferative or plasma cell disorder, and whether patients progressed to another lymphoproliferative or plasma cell disorder during clinical follow up. Given the possibility of delayed workups leading to delayed diagnoses, patients were only considered to have a first diagnosis of MGUS if they did not progress to a B-cell lymphoproliferative or plasma cell disorders (including SMM) within 6 months of MGUS diagnosis. Patients with SMM as their first plasma cell disorder diagnosis, or those that progressed to SMM during study follow up, were included in the final clinical SMM study cohort.

**Figures**


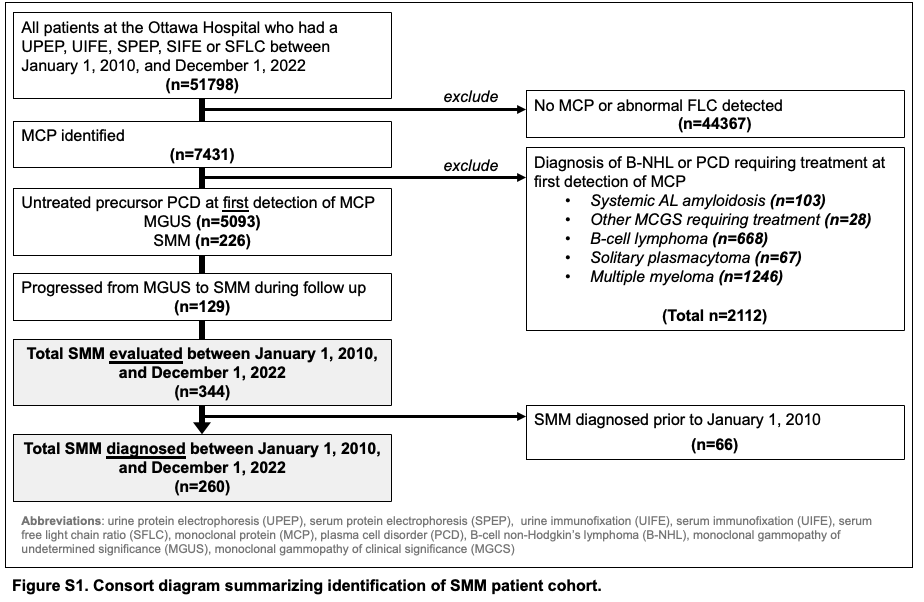


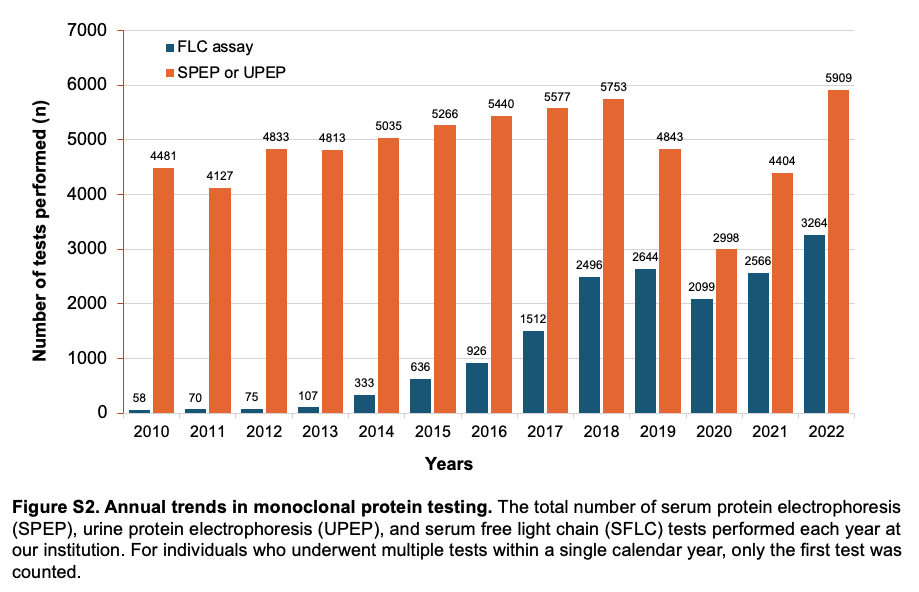


| **Tables**  **Table S1. Age-specific incidence ratios (SIR) of smoldering multiple myeloma** | | | | | |
| --- | --- | --- | --- | --- | --- |
|  | **Champlain LHIN population 2021** (n) | **Observed SMM cases 2016** (n) | **Observed SMM cases 2021** (n) | **Expected SMM cases 2021** (n) | **SIR (95% CI)** |
| Age 40-49 | 174480 | 0 | 2 | 0 | **-** |
| Age 50-59 | 193345 | 2 | 4 | 2 | 2 (0.5-4.4) |
| Age 60-69 | 174690 | 2 | 4 | 2 | 2 (0.5-4.4) |
| Age 70-79 | 114725 | 5 | 11 | 7 | 1.6 (0.8-2.6) |
| Age ≥80 | 62675 | 3 | 5 | 3 | 1.7 (0.5-3.4) |
| **Total age**  **≥40 years** | 719915 | 12 | 26 | 14 | **1.9 (1.2-2.6)** |

*Abbreviations: smoldering multiple myeloma (SMM), Standardized Incidence Ratios (SIR)*

| **Table S2. Annual incidence of SMM among residents of the Champlain LHIN region, stratified by sex and age categories** | | | | | | | | | |
| --- | --- | --- | --- | --- | --- | --- | --- | --- | --- |
|  | **2011** | | | **2016** | | | **2021** | | |
|  | **Champlain LHIN population** (n) | **SMM cases** (n) | **Incidence of SMM** (n per 100,000 people in the reference population) | **Champlain LHIN population** (n) | **SMM cases** (n) | **Incidence of SMM** (n per 100,000 people in the reference population) | **Champlain LHIN population** (n) | **SMM cases** (n) | **Incidence of SMM** (n per 100,000 people in the reference population) |
| **Total population** | 1230655 | 9 | 0.7 | 1292639 | 12 | 0.9 | 1394070 | 26 | 1.9 |
| **High risk SMM** | - | - | - | - | 3^a^ | 0.2 | - | 5^a^ | 0.4 |
| **Male** | 600010 | 5 | 0.8 | 630860 | 2 | 0.3 | 682675 | 15 | 2.2 |
| **Female** | 630645 | 4 | 0.6 | 661780 | 10 | 1.5 | 711395 | 11 | 1.5 |
| **Age ≥40 years** | 617370 | 9 | 1.5 | 670190 | 12 | 1.8 | 719915 | 26 | 3.6 |
| Age 40-49 | 183820 | 0 | 0 | 172285 | 0 | 0 | 174480 | 2 | 1.1 |
| Age 50-59 | 183155 | 0 | 0 | 200190 | 2 | 1 | 193345 | 4 | 2.1 |
| Age 60-69 | 129025 | 2 | 1.6 | 154435 | 2 | 1.3 | 174690 | 4 | 2.3 |
| Age 70-79 | 72900 | 3 | 4.1 | 88070 | 5 | 5.7 | 114725 | 11 | 9.6 |
| Age ≥80 | 48470 | 4 | 8.3 | 55210 | 3 | 5.4 | 62675 | 5 | 8 |

^a^The total population of the Champlain LHIN was used as the reference population to calculate the incidence of high risk SMM.

**Table S3. Prevalence of SMM among residents of the Champlain LHIN over time**

|  | **2011** | | | **2016** | | | **2021** | | |
| --- | --- | --- | --- | --- | --- | --- | --- | --- | --- |
|  | **Champlain LHIN population** (n) | **SMM cases** (n) | **Prevalence of SMM** (n per 100,000 people in the reference population) | **Champlain LHIN population** (n) | **SMM cases** (n) | **Prevalence of SMM** (n per 100,000 people in the reference population) | **Champlain LHIN population** (n) | **SMM cases** (n) | **Prevalence of SMM** (n per 100,000 people in the reference population) |
| **Total population** | **1230655** | **84** | **6.8** | **1292639** | **99** | **7.7** | **1394070** | **159** | **11.4** |
| Male | 600010 | 43 | 7.2 | 630860 | 45 | 7.1 | 682675 | 79 | 11.6 |
| Female | 630645 | 41 | 6.5 | 661780 | 54 | 8.2 | 711395 | 80 | 11.2 |
| **Aged ≥40 years at follow-up** | **617370** | **83** | **13.4** | **670190** | **99** | **14.8** | **719915** | **157** | **21.8** |
| Age 40-49 | 183820 | 2 | 1.1 | 172285 | 1 | 0.6 | 174480 | 6 | 3.4 |
| Age 50-59 | 183155 | 11 | 6 | 200190 | 16 | 8 | 193345 | 20 | 10.3 |
| Age 60-69 | 129025 | 19 | 14.7 | 154435 | 23 | 14.9 | 174690 | 36 | 20.6 |
| Age 70-79 | 72900 | 23 | 31.6 | 88070 | 25 | 28.4 | 114725 | 50 | 43.6 |
| Age ≥80 | 48470 | 28 | 57.8 | 55210 | 34 | 61.6 | 62675 | 45 | 71.8 |

*Abbreviations: smoldering multiple myeloma (SMM*), local health integration networks (LHIN)
